# The Effect of Electrically Induced Cycling and Nutritional Counseling on Cardiometabolic Health in Upper and Lower Motor Neuron Chronic Spinal Cord Injury: Dual Case Report

**DOI:** 10.4172/2376-0281.1000336

**Published:** 2019-01-12

**Authors:** David R Dolbow, Daniel P Credeur, Jennifer L Lemacks, Mujtaba Rahimi, Dobrivoje S Stokic

**Affiliations:** 1 Physical Therapy Program, William Carey University, Hattiesburg, MS, USA; 2 School of Kinesiology and Nutrition, University of Southern Mississippi, Hattiesburg, MS, USA; 3 Biomedical Sciences, William Carey University, Hattiesburg, MS, USA; 4 Center for Neuroscience and Neurological Recovery, Methodist Rehabilitation Center, Jackson, MS, USA

**Keywords:** Functional electrical stimulation, Upper motor neuron injury, Lower motor neuron injury, Cardiometabolic health, Spinal cord injury

## Abstract

**Introduction:**

Various therapies have been utilized to improve cardiometabolic health after spinal cord injury (SCI), including Functional Electrical Stimulation (FES) cycling. Typically, FES is used in SCI cases resulting from Upper Motor Neuron Injury (UMN-SCI). However, it has been reported that FES may improve muscle torque and functional mobility in individuals with Lower Motor Neuron Injuries (LMN-SCI) but potential effects on cardiometabolic health have not been studied before. Thus, this study examined the cardiometabolic health response to FES cycling combined with nutritional counseling in two individuals with chronic SCI; one person with LMN-SCI and one with UMN-SCI.

**Case Presentation:**

Body composition, vascular stiffness, and glucose deposition were assessed before and after participation in the FES cycling and nutritional counseling program. Despite the decrease in body mass in the case of LMN-SCI but not UMN-SCI, the fat mass-to-lean mass ratio in the lower limbs and trunk increased +4% and +8% respectively, in the former and decreased −10% and −8% respectively in the latter. Both subjects decreased markers of central vascular stiffness (AIx@75, reflection magnitude) as well as blood glucose and HbA1c levels, however, the changes were greater in the case of UMN-SCI.

**Discussion:**

This dual case study provides only a partial support for the use of FES cycling alone or in combination with nutritional counseling for improving cardio metabolic health in LMN-SCI, however modest decreases in glucose and vascular stiffness warrant further investigations.

## Introduction

Various therapies have been studied and utilized to improve cardiometabolic health after spinal cord injury (SCI) [1], including Functional Electrical Stimulation (FES) cycling [2–4]. The reported benefits of FES-cycling on cardiometabolic health [5] include increased lean-to-fat-mass ratio [6–10], increased blood flow and vasoreactivity in paralyzed legs [11], and improvements in glucose disposal [6,12]. However, FES is typically used in cases of spastic paralysis resulting from Upper Motor Neuron (UMN) injury where, in addition to orthodromic muscle activation, intact peripheral nerves can carry an electrical stimulus upstream to spinal reflex centers and thereby produce descending signals to evoke reflex-mediated muscle contractions. In cases with a Lower Motor Neuron (LMN) injury affecting the lumbosacral region, and the associated partial or total loss of peripheral nerves, it is more difficult or impossible to elicit both direct and indirect muscle contractions [13]. As a result, the study of the effects of FES cycling in individuals with LMN injuries has been limited.

Previous reports do indicate potential health benefits of FES for persons with LMN injury. For example, 80 sessions of FES 5 days/week, 60 minutes each, increased lower extremity muscle torque and improved functional mobility [14]. Similarly, one case report demonstrated that 14 sessions of FES increased ankle function in a person who sustained a sacral fracture and nerve root injury [4]. These reports recommended further study using FES for improving motor function in those with peripheral nerve injuries [4,14]. However, it is currently unknown whether FES training can also improve cardiometabolic health in cases of spinal cord LMN injuries.

As such, the purpose of this report was to examine the cardiometabolic health response (i.e., body composition, muscle architecture, autonomic function, and vascular stiffness) to FES cycling combined with nutritional counseling in two individuals with chronic SCI; one person with an LMN injury (LMN-SCI), and one with an UMN injury (UMN-SCI). Nutrition counseling was included because nutrition consumption has been reported as one factor that may obscure the expected body composition and metabolic profile adaptations anticipated from FES training [15].

### Dual Case Presentation

This dual case report evolved from our prospective pilot study examining the effects of combined FES cycling and nutritional counseling in individuals with SCI. After determining that a prospective subject suffered LMN-SCI, while all other participants suffered from UMN-SCI, it was decided to offer the individual to participate in the program as a case study given the reports of motor improvements after FES in this population [4,14]. For proper comparison, this individual was matched post-hoc with the UMN-SCI subject of similar age, motor severity of SCI, and time since the injury. A limitation in the match process was that we were not able to match participants by gender which is a possible potential confounder.

### Subjects

The subjects’ demographic and injury characteristics are presented in Table 1.

### Procedures

#### High-intensity interval-FES training:

Both subjects participated in high-intensity interval-FES training using the RT 300 device, as described before [16]. Surface electrode placement and FES cycle set-up followed prior published guidelines [17]. In both subjects, FES pulse width was 400 |is and frequency 40 Hz. The stimulation intensity was set individually, as follows.

For the UMN-SCI subject, the FES intensity was selected based on the response to an incremental electrical stimulation until a strong visible muscle contraction was produced. As a result, FES cycling intensity started with 90 mA for quadriceps and hamstring muscle groups and 80 mA for gluteal muscles. The stimulation intensity required to sustain cycling reached the maximum of 140 mA for the quadriceps and hamstring muscles, and 130 mA for gluteal muscles within multiple sessions. During the high-intensity cycling intervals, 100% stimulation intensity was used, which decreased by 50% in the low-intensity intervals. High and low intervals alternated every 30 seconds, over 30 minutes of FES cycling. In the LMN-SCI subject, no visible or palpable muscle contraction could be evoked in the stimulated muscles, either pre-, during, or post-FES sessions, thus the maximal intensity (140 mA) was selected for all muscles from the outset.

FES cycling was set at a cadence of 35 rpm with an initial resistance of 0.5 Nm. For the UMN-SCI subject, the resistance gradually increased during the high-intensity intervals up until the cycling cadence could no longer be sustained without motor support (1.175 Nm). Since FES in the LMN-SCI could not achieve the target cycling cadence against 0.5 Nm, the motor provided support during all sessions throughout the program. Resistance for the low-intensity intervals was maintained at 0.5 Nm for both subjects.

#### Nutritional counseling:

Both subjects received a 30 minute nutritional counseling session over the phone once per week for the first 8 weeks. A registered dietitian conducted the sessions and tailored the content to the individual needs. The focus of counseling was on making healthy food choices without encouraging caloric restrictions.

#### Assessment of body composition and musculoskeletal health:

Body composition was assessed using a Lunar Prodigy Advance Dual-Energy X-Ray Absorptiometry (DXA) scanner (General Electric, Madison, WI). Measurements of the total body and regional (legs) percent body fat, fat mass, and lean mass were made by the DXA Lunar software version 10.5. The DXA scans were performed prior to and after the FES training program (Table 2).

Muscle architecture of the right vastus lateralis muscle (VL) was evaluated using B-mode ultrasound (GE, Logiq-P5), as previously described [18]. Subcutaneous fat thickness (Sub-Q) was defined as the distance between the skin surface and superficial fascia of the VL. VL muscle thickness was defined as the distance between the superficial fascia and the deep aponeurosis, with total Thigh Thickness (TT) defined as the distance between the skin surface and deep aponeurosis.

#### Assessment of cardiometabolic health:

Fasting blood glucose (Reli-On) and glycosylated hemoglobin-HbA1c (PTS Diagnostics, A1c Now+) were obtained *via* a finger stick, and calculated using commercially available testing devices. The cardiac autonomic function was assessed from Electrocardiogram (ECG) to determine Heart Rate (HR) and H-R Variability (HRV) according to guidelines [19]. Indices of aortic vascular stiffness were determined *via* pulse wave analysis (SphygmoCor XCEL) [20]. To do this, brachial artery pressure waveforms were obtained from a standard arm cuff *via* the oscillometric method and transformed into aortic pressure waves using a validated transfer function [21], from which the following central hemodynamic measures were derived: central mean arterial pressure (cMAP), augmentation index normalized to heart rate at 75 bpm (AIx@75), and wave reflection magnitude (RM).

## Results

The LMN-SCI subject completed 21 sessions over 15 weeks (1.4/ wk). The UMN-SCI subject completed 22 FES cycling sessions over a 9.5-week period (2.3/wk). Both subjects completed all 8 nutritional counseling sessions over the phone.

### Cardiometabolic health response

#### Body composition:

The LMN-SCI subject decreased body weight by ~2.7%, from 110.6 to 107.6 kg, but increased in body fat % from 49.9 to 51.3%. The UMN-SCI subject had only a 0.46 kg decrease in body weight (76.6–76.1 kg), and a decrease in body fat% from 54.9 to 53.2%. The greatest difference between the subjects following FES training was in the fat mass-to-lean mass ratio in the lower limbs and trunk, which increased in the LMN-SCI subject (+4% and +8%, respectively) but decreased in the UMN-SCI subject (−10% and −8%, respectively).

#### Muscle architecture:

The LMN-SCI subject exhibited an increase in SubQ (+49%; from 1.9 – 2.8 mm), but a decrease in VL muscle thickness (−28%; 2.9 to 2.1 mm), with little change occurring in TT thickness (+2%; 4.8 to 4.9 mm). The UMN-SCI subject, however, exhibited a decrease in SubQ (−14%; from 2.4 to 2.1 mm), VL thickness (−21%; 2.8 to 2.2 mm), and TT thickness (−17%; 5.2 to 4.3 mm).

#### Metabolic health:

Both subjects demonstrated a decrease in blood glucose and HbA1c (LMN-SCI: blood glucose from 119 to 88 mg/dL, HbA1c from 4.8 to 4.6%; UMN-SCI: blood glucose from 113 to 98 mg/ dL, HbA1c from 5.5 to 5.2%).

#### Cardiovascular health:

Resting HR changed minimally in the UMN-SCI subject (58 to 57 bpm) but increased in the LMN-SCI subject (85 to 98 bpm) following FES training. In both subjects, similar decreases were found in R-R variability (SDNN: −52% on average) and the high-frequency component (HF: −86% on average). However, LF-to-HF component ratio increased in UMN-SCI (LF/HF: 0.9 to 2.2AU) but decreased in LMN-SCI (LF/HF: 4.1 to 2.2AU). For pulse wave analysis, cMAP increased in both LMN-SCI (79 to 86 mmHg) and UMN-SCI (99 to 103 mmHg) subjects but the AIx@75 decrease was less pronounced in LMN-SCI (−0.5 to −2.5%) and UMN-SCI (40.5 to 29%) subject. Reflection magnitude decrease was also greater in UMN-SCI (14%) than LMN-SCI subject (5%).

## Discussion

Although the LMN-SCI subject lost 3 kg of body mass, this did not translate into a more favorable body composition since the fat-mass- to-lean-mass ratio increased following the intervention. We speculate that the loss of body mass in this subject was due to the nutritional counseling because FES was unable to elicit muscle contractions and thereby provide sufficient physical activity to prevent muscle mass loss. In the UMN-SCI subject, however, FES induced muscle contractions and thus provided meaningful exercise, which is likely the reason for an increase in lean mass and a more favorable fat-mass-to-lean-mass ratio. At the same time, both subjects modestly improved glucose metabolism as assessed by blood and HbA1c levels.

Regarding cardiovascular health outcomes, changes in autonomic function (i.e., low-to-high frequency component ratio) appeared to favor greater sympathetic support in the case of UMN-SCI, but potentially, greater parasympathetic activity in LMN-SCI. Both subjects exhibited a decrease in markers of central vascular stiffness (i.e., AIx@75 and RM), however, the changes were more pronounced in the case of UMN-SCI.

Collectively, our contrasting results provide only a partial support for the use of FES cycling alone or in combination with nutritional counseling to improve markers of cardiometabolic health (i.e., body composition) in individuals with LMN injuries. The benefits reported in the case of LMN-SCI, namely, a modest decrease in glucose and vascular stiffness, could be attributed to passive movements achieved *via* motor support. It is important to note that our FES device (RT300) does not allow the use of longer pulse widths and higher frequencies as reportedly used in the previous LMN-SCI study 14.

### Limitations

This is a report on two individuals with SCI, thus, the results cannot be generalized to the broader SCI population.

## Conclusion

FES cycling, used in conjunction with nutritional counseling, may be effective in reducing and even reversing some of the deteriorative metabolic and cardiovascular changes that occur in individuals with long-standing UMN-SCI. Further study is needed to explore the potential benefits of passive cycling on glucose deposition and vascular stiffness in individuals with LMN-SCI.

## Figures and Tables

**Table 1:** Demographic and injury characteristics of SCI subjects.

Subject	Age	Sex	Race	Injury Level	AIS Grade	Time post-SCI
UMN-SCI	34 years	Female	Caucasian	C6	B	10 years
LMN-SCI	34 years	Male	Caucasian	T11	A	8 years

Note: SCI: Spinal Cord Injury; UMN: Upper Motor Neuron; LMN: Lower Motor Neuron; AIS: American Spinal Injury Association Impairment Scale.

**Table 2:** Body composition changes.

Subject	Δ Wt (kgs)	Δ BF%	Δ Fat-Lean ratio Legs	Δ Fat-Lean ratio Trunk
UMN-SCI	< −1%	−3.1%	−10%	−8%
LMN-SCI	−2.7%	+2.8%	+4%	+8%

Note: SCI- Spinal Cord Injury; UMN: Upper Motor Neuron; LMN: Lower Motor Neuron, Wt: Weight, BF%: Body Fat Percentage, Δ: Change.
